# An α-synuclein decoy peptide prevents cytotoxic α-synuclein aggregation caused by fatty acid binding protein 3

**DOI:** 10.1016/j.jbc.2021.100663

**Published:** 2021-04-20

**Authors:** Naoya Fukui, Hanae Yamamoto, Moe Miyabe, Yuki Aoyama, Kunihiro Hongo, Tomohiro Mizobata, Ichiro Kawahata, Yasushi Yabuki, Yasuharu Shinoda, Kohji Fukunaga, Yasushi Kawata

**Affiliations:** 1 Department of Chemistry and Biotechnology, Faculty of Engineering/Graduate School of Engineering, Tottori University, Tottori, Japan; 2 Department of Biomedical Science, Institute of Regenerative Medicine and Biofunction, Graduate School of Medical Science, Tottori University, Tottori, Japan; 3 Center for Research on Green Sustainable Chemistry, Tottori University, Tottori, Japan; 4 Department of Pharmacology, Graduate School of Pharmaceutical Sciences, Tohoku University, Sendai, Japan

**Keywords:** α-synuclein, amyloid fibrils, fatty acid binding protein 3, protein aggregation, synuclein-fatty acid binding protein 3 complex, αSyn, α-synuclein, αSynP, synthetic peptide derived from α-synuclein, AFM, atomic force microscopy, ANS, 1-anilinonaphtalene-8-sulfonate, CD, circular dichroism, FABP3, fatty acid binding protein 3, FBS, fetal bovine serum, FRET, fluorescence resonance energy transfer, GFP, green fluorescent protein, His6-FABP3, FABP3 with N-terminal His6 tag, JC-1, 5, 5’, 6, 6’-tetrachloro-1, 1’, 3, 3’-tetraethylbenzimidazolylcarbocyanine iodide, MEM, minimal essential medium, MTS, 3-(4,5-dimethylthiazol-2-yl)-5-(3-carboxymethoxyphenyl)-2-(4-sulfophenyl)-2H-tetrazolium, N2a, neuro2a, PD, Parkinson’s disease, PUFA, polyunsaturated fatty acid, QCM, quartz crystal microbalance, TEM, transmission electron microscopy, Thio-T, Thioflavin-T

## Abstract

α-synuclein (αSyn) is a protein known to form intracellular aggregates during the manifestation of Parkinson’s disease. Previously, it was shown that αSyn aggregation was strongly suppressed in the midbrain region of mice that did not possess the gene encoding the lipid transport protein fatty acid binding protein 3 (FABP3). An interaction between these two proteins was detected *in vitro*, suggesting that FABP3 may play a role in the aggregation and deposition of αSyn in neurons. To characterize the molecular mechanisms that underlie the interactions between FABP3 and αSyn that modulate the cellular accumulation of the latter, in this report, we used *in vitro* fluorescence assays combined with fluorescence microscopy, transmission electron microscopy, and quartz crystal microbalance assays to characterize in detail the process and consequences of FABP3–αSyn interaction. We demonstrated that binding of FABP3 to αSyn results in changes in the aggregation mechanism of the latter; specifically, a suppression of fibrillar forms of αSyn and also the production of aggregates with an enhanced cytotoxicity toward mice neuro2A cells. Because this interaction involved the C-terminal sequence region of αSyn, we tested a peptide derived from this region of αSyn (αSynP130-140) as a decoy to prevent the FABP3–αSyn interaction. We observed that the peptide competitively inhibited binding of αSyn to FABP3 *in vitro* and in cultured cells. We propose that administration of αSynP130-140 might be used to prevent the accumulation of toxic FABP3-αSyn oligomers in cells, thereby preventing the progression of Parkinson’s disease.

A pathological hallmark of Parkinson’s disease (PD) is the aggregation/deposition of insoluble protein in the cell known as Lewy bodies (1, 2). The main protein component of Lewy bodies is α-synuclein (αSyn), an intrinsically unfolded polypeptide composed of 140 amino acid residues, that is highly expressed in neurons. The amino acid sequence of αSyn is distinguished by three characteristic domains: an amphiphilic N-terminal domain (amino acid residue number; 1–65) enriched in positively charged lysine residues (3), a central domain (the NAC domain; 66–95) which contains many hydrophobic amino acid residues, and a negatively charged C-terminal domain (96–140) (4). In the N-terminal and the NAC domains, there are seven incomplete KTKEGV sequence repeats (5). In *in vitro* experiments, αSyn has been shown to form regular, β-sheet–enriched filamentous aggregates that specifically bind to the fluorescent dye Thioflavin-T (Thio-T) (6). Numerous extensive studies have shown that during the pathological progression of PD, aggregates formed by αSyn display cytotoxicity. Detailed experiments have demonstrated that the specific form of aggregated αSyn that is most toxic to cells is a soluble, oligomeric form that is formed before the maturation of higher order, insoluble fibrils that are eventually detected in pathological screens (7, 8, 9, 10). In this context, the Lewy bodies that are detected as a hallmark of PD may be regarded as an evolved method to “isolate and contain” cytotoxic soluble oligomers. Details regarding the specific correlation between cellular toxicity and various forms of αSyn are still unclear, however, especially when the highly heterogeneous environment within typical eukaryotic cells are likely to alter various factors of this relationship.

Previously, it was found that polyunsaturated fatty acids (PUFAs) are capable of binding to αSyn and accelerating the oligomerization of this protein (11). The N-terminal region of αSyn was implicated in this interaction with PUFAs (12). This interaction between αSyn and PUFAs may also be biologically relevant, because PUFAs bound to αSyn were transported into cells (13), causing an increase in the rate of cellular import of PUFAs (14). Typically, the import of fatty acid and PUFAs into the cell is mediated by a small group of transport proteins known as the fatty acid binding proteins (FABPs) (15). In mammals, ten FABP subtypes have been characterized (16, 17) and are generally grouped according to the degree of protein expression in various organs and cell types, although more detailed characterizations have confirmed the expression of certain FABPs in multiple locales (18). The sequence homology between FABP subtypes ranges between 20 and 70%; however, the family is characterized by a common tertiary structural motif which consists of a central β-barrel structure composed of ten β-strands and two α-helices in the N-terminal portion. Fatty acid molecules are recognized and bound by the β-barrel motif. Each FABP subtype shows binding preferences toward different fatty acids; cardiac H-FABP (FABP3) preferentially binds *n-6* polyunsaturated fatty acids, epidermal E-FABP (FABP5) recognizes saturated fatty acids, and brain B-FABP (FABP7) recognizes *n-3* polyunsaturated fatty acids such as α-linolenic acid, docosahexaenoic acid, and eicosapentaenoic acid (19). In brain tissue, all three of these FABP subtypes are expressed (20, 21). Experiments have shown that serum levels of FABP3 were significantly higher in patients of dementia with Lewy bodies and PD compared with the levels in Alzheimer's disease patients (22). The structure of FABP3 is characterized by a β-barrel structure within which a cluster of water molecules reside. This cluster of water molecules acts as an interface to bind various PUFA molecules, in cooperation with numerous hydrophobic amino acid side chains. FABP3 is also capable of binding to various hydrophobic molecules *via* these amino acid side chains. An amphipathic fluorescence probe, 1-anilino-8-napthalene sulfonate (ANS), has also been utilized to detect PUFA binding to FABP3 (23, 24, 25).

Interestingly, when mice lacking the FABP3 gene were subjected to treatment with 1-methyl-4-phenyl-1,2,3,6-tetrahydropyridine to induce PD-like symptoms, cells sampled from midbrain lacked the characteristic accumulation of αSyn that could typically be observed in WT mice. This finding suggested the existence of a strong relationship between αSyn aggregation and FABP3 (26), specifically, a preventive effect by FABP3 toward αSyn aggregation.

To confirm this relationship between an intrinsically unfolded polypeptide and a fatty acid binding protein, in this article, we report our results of our experiments regarding the interactions between αSyn and FABP3. We confirm that αSyn indeed interacts specifically with the apo form of FABP3, and this interaction causes changes in the fibrillation behavior of the former polypeptide. Furthermore, our experiments revealed that αSyn and FABP3 interactions are also present in cultured cells, as monitored by fluorescence resonance energy transfer (FRET) experiments, and that this interaction results in an increase in cytotoxicity. We also succeeded in identifying the specific site in αSyn that is required for this interaction and, by using this information, propose a method to control this interaction to neutralize the deleterious effects of FABP3.

## Results

### Amyloid fibril formation of αSyn is suppressed by FABP3 binding *in vitro*

#### Addition of FABP3 alters the *in vitro* fibril forming abilities of αSyn

**Figure 1:**
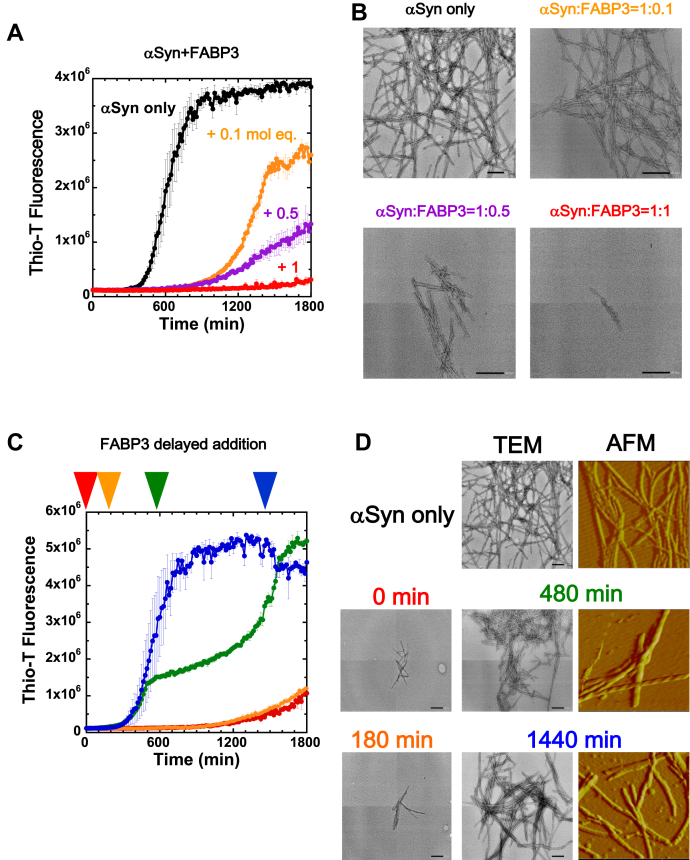
**Effects of FABP3 on the fibril forming tendencies of αSyn.***A*, Thio-T fluorescence traces of αSyn (69 μM) amyloid fibril formation. *Black traces* denote experiments without added FABP3, and the *orange*, *magenta*, and *red traces* denote αSyn fibril formation in the presence of 0.1-mol equivalent, 0.5-mol equivalent, and equimolar concentrations of FABP3, respectively. Error bars denote standard error values calculated from three separate samples. *B*, TEM images of samples depicted in (*A*). Scale bars indicate 200 nm. The legend colors correspond to the notation used in (*A*). *C*, effects of delayed addition of FABP3 to fibril-forming αSyn reaction mixtures. *Red*, *orange*, *green*, and *blue arrows* denote delayed addition of FABP3 after an initial incubation of 0, 180, 480, and 1440 min, respectively. *D*, TEM and AFM images of samples shown in panel (*C*) after completion of the assay (1800 min). Shown at *top* are reference TEM [same image of the “*αSyn only*” in panel (*B*)] and AFM images for αSyn fibrils formed in the absence of FABP3. AFM images for the 0 min and 180 min delayed addition samples were not obtainable because of the scarcity of fibril samples. αSyn, α-synuclein; AFM, atomic force microscopy; FABP3, fatty acid binding protein 3; TEM, transmission electron microscopy; Thio-T, Thioflavin-T.

A previous study has demonstrated that in mammalian cells, co-expression of FABP3 and αSyn results in increased intracellular aggregation of the latter (26). To clarify the relationship between these two proteins, we show in Figure 1 *in vitro* fibril-forming reactions of αSyn that compare the effects of adding FABP3 to the experiment. In the absence of FABP3, αSyn formed amyloid fibrils after an initial lag phase under standard agitating conditions as monitored with Thio-T (Fig. 1*A*). These fibrils could be observed in transmission electron microscopy (TEM) images as regular twisted fibrils that were about 10∼20 nm in width (Fig. 1*B*). Addition of increasing amounts of FABP3 to the reaction caused a gradual elongation of the initial lag phase, as well as a decrease in the cumulative Thio-T signal at the end of the assay (Fig. 1*A*), signifying a decrease in the amount of fibrils formed during the experiment. As shown in Figure 1*B*, TEM images of these reactions showed that at higher FABP3 concentrations, it became increasingly more difficult to obtain images of fibrillar material. A closer look suggests that the fibril forms that were observed in experimental samples containing higher concentrations of FABP3 were also slightly different in form and tended toward shorter fibers. The addition of FABP3 served to suppress the formation of αSyn fibrillar structures that would be normally formed under the conditions that we used, presumably through direct interaction with αSyn. Importantly, no significant suppressive effects in fibrillation of αSyn were observed when FABP3 (pI 7.8) was replaced with bovine serum albumin (pI 4.7) or lysozyme (pI 9.8).

Next, we performed delayed-addition experiments of FABP3 to αSyn to determine if there was a threshold to the time frame in which FABP3 could assert its suppressive effects toward αSyn fibrillation. As shown in Figure 1*C*, adding FABP3 to the mixture during the initial lag phase of fibrillation resulted in effective suppression of the Thio-T signal, whereas adding FABP3 during the extension phase of the reaction resulted in only partial suppression. Interestingly, the addition of FABP3 during the fibril extension phase initially resulted in a strong suppression of further fibril formation, but this effect was eventually overcome; the increase in fluorescence signal at the end of the assay was equal to the signal seen in the absence of FABP3 (Fig. 1*C*, compare *blue* and *green* traces at *t* = 1800 min). This indicated that the effects of FABP3 on αSyn fibrillation became gradually weaker as the reaction progressed, and once extensive formation of fibrils had begun, the suppressive effects of FABP3 were eventually superseded by the tendency of αSyn to form fibrils. Adding FABP3 at the end of the reaction caused minimal change to the Thio-T fluorescence signal, suggesting that FABP3 is incapable of resolubilizing αSyn fibrils once they have formed. These effects of FABP3 were also reflected in the morphology of fibrils formed under these various conditions. As shown in Figure 1*D*, when FABP3 was added during the lag phase (*t* ≦ 180 min), the fibrils formed were shorter in morphology and more sparsely dispersed in the TEM observation field. In contrast, for samples where FABP3 was added in the latter portion of the reaction, there were no detectable differences in the width and height of the fibrils observed in the TEM and atomic force microscopy (AFM) images (Table 1).

**Table 1:** Average height and width values for fibrils derived from AFM images shown in Figure 1*D* (number of measurements: 6)

Sample	Height (nm)	Width (nm)
αSyn fibril	7.4 ± 0.1	59 ± 1.2
αSyn; FABP3 addition at 480 min	5.7 ± 0.1	55 ± 1.4
αSyn; FABP3 addition at 1440 min	8.0 ± 0.2	60 ± 1.3

αSyn, α-synuclein; AFM, atomic force microscopy; FABP3, fatty acid binding protein 3.

#### Structural and functional characterization of the αSyn–FABP3 complex

**Figure 2:**
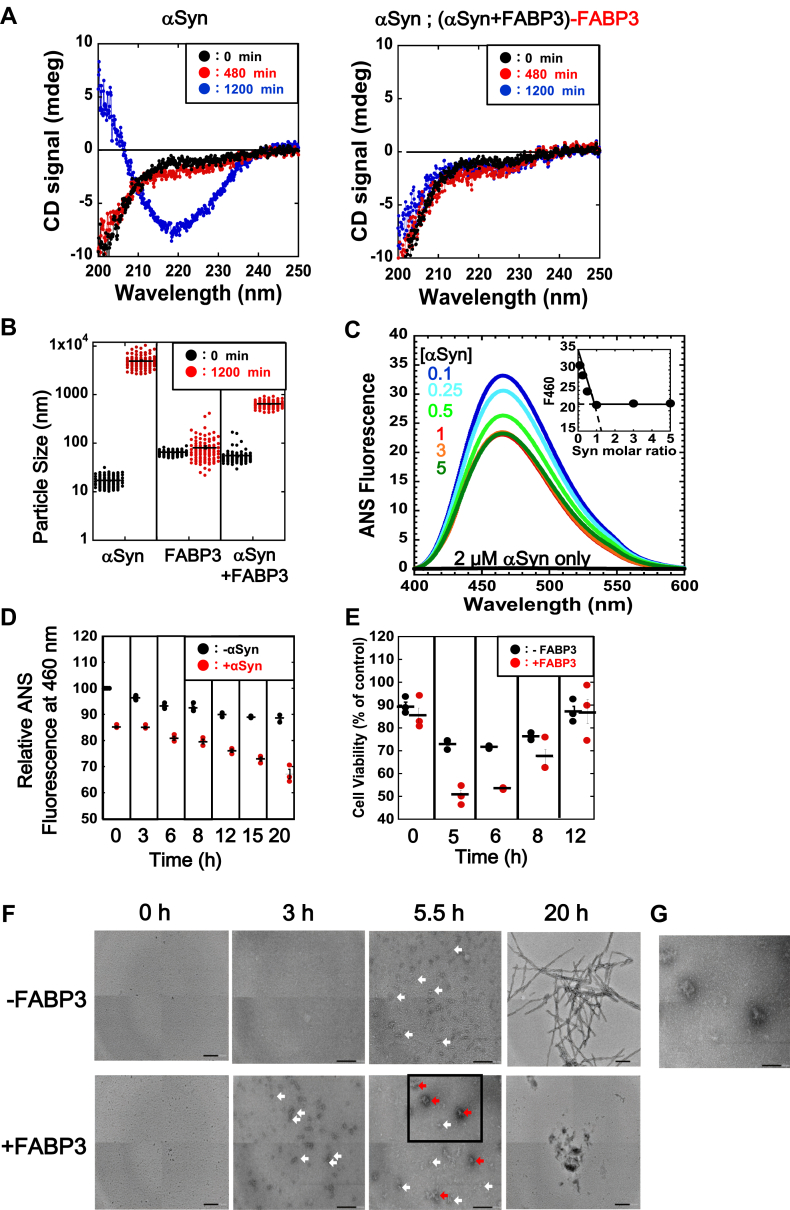
**Structural analysis of αSyn fibrils formed in the presence of FABP3.***A*, CD spectral comparisons of αSyn fibrils formed in the presence (*right panel*) and absence (*left panel*) of FABP3. The *left panel* “*αSyn*” compares the CD spectra of αSyn that form fibrils in the absence of FABP3. *Black*, *red*, and *blue traces* denote spectra taken after an incubation interval of 0, 480, and 1200 min, respectively. The *right panel* “*αSyn; (αSyn+FABP3)-FABP3*” is a comparison of the traces in panel *“αSyn”* after subtraction of CD spectrum of FABP3 from that of αSyn+FABP3 sample, which shows that signal changes in CD attributable to αSyn are negligible when incubated in the presence of FABP3. *B*, dynamic light scattering analysis of αSyn fibril samples formed in the presence and absence of FABP3. *Black dots* denote individual particle sizes for initial samples (before fibrillation), and *red dots* indicate values for samples incubated for 1200 min. Data points for 100 measurements are shown in combination with the mean value denoted by *bars*. *C*, changes in ANS-derived fluorescence for αSyn samples incubated in the presence of increasing concentrations of FABP3. The concentration of αSyn that was added to a fixed concentration of FABP3 (2 μM) is shown as colorized values within the panel. The *inset* shows a plot of the fluorescence emission signal at 460 nm (F460) for each spectrum shown in the main figure as a function of the αSyn concentration added. The plot shows that the decrease in F460 signal saturates at a 1:1 molar ratio of FABP3 to αSyn, suggesting stoichiometric binding. The excitation wavelength was 378 nm. *D*, time-dependent changes in the ANS-derived fluorescence (Em: 460 nm) for FABP3 incubated with αSyn, indicating the in the presence of αSyn, the ANS fluorescence signal for FABP3 decreases over time. *E*, toxicity analysis by MTS assay of αSyn fibril samples incubated in the presence of FABP3 *in vitro*. αSyn was incubated to the intervals indicated in the ordinate to induce fibril formation. FABP3 was added to the *red samples*. Aliquots of these mixtures were added to cultures of N2a cells to assess cellular toxicity after a 24-h incubation, as described in the Experimental procedures section. *F*, TEM images of αSyn and FABP3 samples that were incubated for 0, 3, 5.5, and 20 h. The “0 h”, “5.5 h”, and “20 h” samples observed were obtained from aliquots of samples used in the MTS assays shown in panel (*E*). Small (50–80 nm in diameter) and larger (90–150 nm in diameter) aggregate molecules that were observed in the samples (notably, in the 3 h and 5.5 h samples) are indicated by the *white* and *red arrows* respectively. Notably, the larger aggregates were only observed in the αSyn+FABP3 samples incubated for 5.5 h (“+FABP3, 5.5 h”). Scale bars indicate 200 nm. *G*, localized reimaging of the area indicated by the *square* in (*F*), at 80K magnification. The scale bar in this image indicates 100 nm. αSyn, α-synuclein; ANS, 1-anilino-8-napthalene sulfonate; CD, circular dichroism; FABP3, fatty acid binding protein 3; MTS, 3-(4,5-dimethylthiazol-2-yl)-5-(3-carboxymethoxyphenyl)-2-(4-sulfophenyl)-2H-tetrazolium.

Building upon the results that indicated a reduced fibrillation of αSyn in the presence of FABP3 in Figure 1, we next probed for details regarding the structural and functional consequences of αSyn–FABP3 binding that result in the suppression of αSyn fibrils. In Figure 2*A*, we compare the circular dichroism (CD) spectra of αSyn samples allowed to form fibrils in the absence and presence of an equimolar concentration of FABP3. Because FABP3 was characterized by a strong negative CD absorbance in the far UV region that reflects its secondary structural composition, the comparisons for αSyn structure must be performed after the contribution of FABP3 to the overall spectra are subtracted. As shown in Figure 2*A* (left), in the absence of FABP3, the CD spectra of αSyn is reflective of a structural transition between an intrinsically disordered protein (the original state of αSyn) and a β-structure-rich fibril state with a negative CD absorption maximum at around 218 nm (Fig. 2*A* [left], “αSyn”, compare *black* and *blue* traces). In the presence of equimolar FABP3 (Fig. 2*A* [right]); however, this transition was not immediately apparent; comparison of the CD spectra after the FABP3 component has been subtracted indicated that the CD signal of αSyn remained largely unchanged for the duration of the fibril extension experiment, suggesting that the presence of FABP3 served to maintain αSyn in its largely disordered state (Fig. 2*A* (right), “αSyn; (αSyn+FABP3)-FABP3”). These findings were also supported by analysis of these results using BeStSel (27), as summarized in Table 2.

**Table 2:** BeStSel secondary structure estimation from CD spectra shown in Figure 2*A*

Estimated secondary structure content (%)	αSyn (0 min)	αSyn = (αSyn +FABP3)- FABP3 (0 min)	αSyn (480 min)	αSyn = (αSyn +FABP3)- FABP3 (480 min)	αSyn (1200 min)	αSyn = (αSyn +FABP3)- FABP3 (1200 min)
Helix	0	0	2.6	1.1	3.9	1.8
Parallel	0	0	0	0	41.5	0
Antiparallel	29.2	40.5	32	47.5	44.2	33.1
Turn	13.6	11.8	15.5	11.3	0	17.6
Others	57.1	47.7	49.8	40.1	10.4	47.5

αSyn, α-synuclein; CD, circular dichroism; FABP3, fatty acid binding protein 3.

The binding of FABP3 to αSyn also resulted in a measurable decrease in the size of aggregates that were formed during the fibril forming reaction, as shown in Figure 2*B*. Dynamic light scattering analysis showed that while αSyn alone formed particles (fibrils) with an average particle size of 6 μm in the absence of FABP3 after a 1200 min incubation, in the presence of FABP3, the average size of the particles decreased to almost 1/10 of this value to 0.63 μm. This value, which was greater than the average particle size (50 nm) for FABP3 alone during an equivalent incubation, suggested that the binding of FABP3 to αSyn was successful in suppressing the large aggregates (fibrils) that the latter protein would tend to form under similar conditions. The effects of FABP3 on αSyn fibrillation seemed to bind and form a stable complex that would prevent the formation of larger oligomers that would eventually lead to insoluble fibrils.

In an accompanying experiment, we demonstrated that the interaction between αSyn and FABP3 also results in changes in the surface hydrophobicity of FABP3 as well. In Figure 2*C*, we show changes in the ANS fluorescence spectra of FABP3 samples incubated in the presence of increasing concentrations of αSyn (25). As shown in the figure, addition of increasing concentrations of αSyn to FABP3 results in the gradual decrease of ANS-derived fluorescence. As shown in the inset to Figure 2*C*, the decrease in fluorescence signal was retarded abruptly when more than a 1:1 concentration of αSyn was added to FABP3 samples, which suggested that these two proteins were binding stoichiometrically under these conditions. The time course of this decrease in fluorescence revealed additional insights. As visualized in Figure 2*D*, when we monitored the changes in ANS-derived fluorescence of αSyn–FABP3 complexes over a period of 20 h, we observed that the decrease in ANS fluorescence (attributed to a decrease in the surface hydrophobicity of FABP3 being occluded by the binding of αSyn) proceeded gradually over this time period. This additional insight suggested that, once formed, the αSyn–FABP3 complex matured into a different soluble form characterized as shown in Figure 2*B* ("αSyn+FABP3" at 1200 min), one with a decreased hydrophobic surface area and a slightly larger size. This insight was an additional hint to the potential consequences that such a conformational change may pose to the cellular function of these two proteins.

Do the complexes formed by αSyn and FABP3 display toxicity toward cells? To answer this question, we performed cell toxicity assays by adding various fractions of αSyn sampled during the course of fibrillation to mammalian cell cultures. The toxicity of aggregated αSyn species is typically highest for the so-called “oligomeric” forms that are most abundant immediately before the extension phase of the reaction (7, 8, 9, 10); the results in Figure 2*E*, “-FABP3”, support this prior finding. Interestingly, a stronger toxicity was observed for the "αSyn + FABP3" sample at 5 to 6 h compared with αSyn only (Fig. 2*E*, compare “+FABP3” and “-FABP3”). This result suggested that the binding of FABP3 to αSyn does result in the formation of alternate oligomeric forms with stronger cellular toxicities. Formation of the αSyn–FABP3 complex results in a suppression of the mature fibril form of αSyn, possibly through diversion of αSyn–FABP3 complex to an alternate form with higher toxicity. To observe the details of the oligomeric structures, we measured TEM images for αSyn and αSyn–FABP3 complex samples incubated for various intervals (Fig. 2*F*). As shown in Figure 2*F*, we observed small aggregates that were 50 to 80 nm in diameter in samples of αSyn incubated singly for 5.5 h and samples of αSyn–FABP3 incubated for 3 and 5.5 h, respectively. Additionally, in the images of αSyn–FABP3 complex incubated for 5.5 h, we observed larger sized oligomers 80 to 150 nm in diameter, which may also correspond to toxic species of αSyn–FABP3 complex.

To summarize, αSyn and FABP3 form a αSyn–FABP3 complex *in vitro* at a 1:1 molar ratio, and this complex effectively prevents αSyn fibrillation. Over time, this complex transiently formed a soluble cytotoxic species [(αSyn-FABP3)n oligomer] *in vitro*.

### Binding of αSyn to FABP3: The C-terminal peptide of αSyn binds to the fatty acid-binding pocket of FABP3

#### Identification of FABP3-binding sequence regions of αSyn

We next probed for information regarding the actual binding interactions between αSyn and FABP3, beginning with the determination of the specific sequence region of αSyn that was most directly involved in intermolecular interactions. To achieve this, we prepared three forms of αSyn with specific sequence alterations to the C-terminal sequence region (from residues 130 to 140) of αSyn, because this region has previously been implicated in the formation of αSyn fibrils (28, 29, 30). The variant αSynΔ130-140 (Δ130) is a truncated version of αSyn with the region in question omitted. In the αSyn130-140CF (Charge-Free) variant (130CF), all of the charged sequences in this sequence segment (130-**EE**GYQ**D**Y**E**P**E**A-140, bolded) were substituted with polar, noncharged asparagine residues. In the αSynY133A/Y136A variant (YA/YA), two hydrophobic and aromatic tyrosine residues in this region were substituted with neutral alanine residues. The three variants comprise a survey of the general importance of sequence locale, hydrophilicity, and hydrophobicity with regard to this sequence segment by selectively altering each of these possible contributing factors.

**Figure 3:**
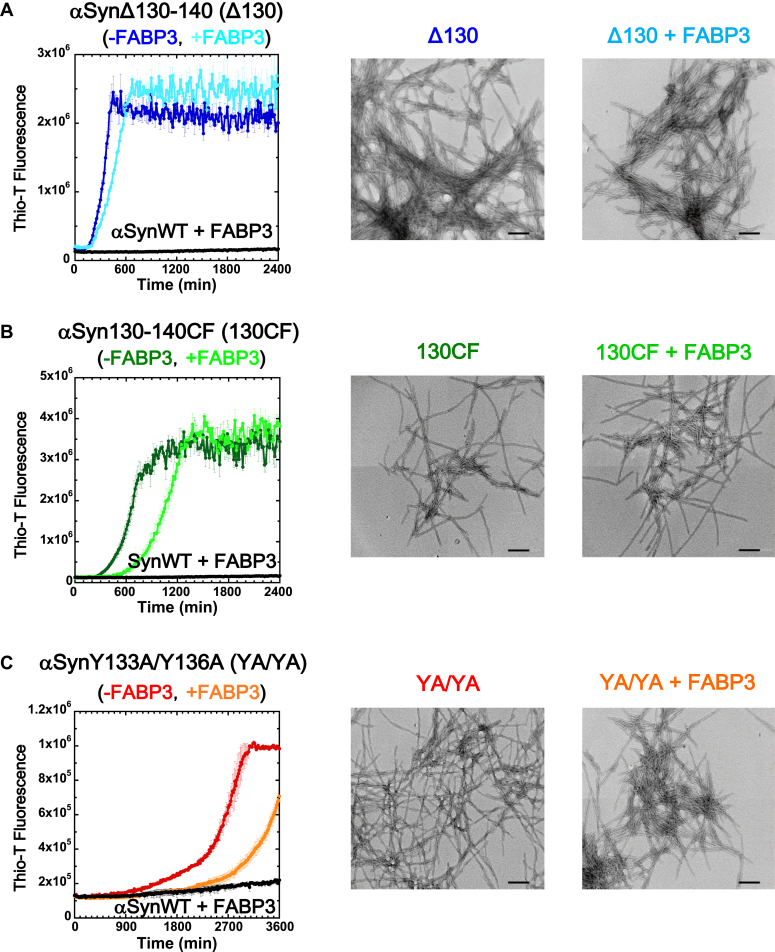
**Involvement of the C-terminus region of αSyn in FABP3 binding.** Three different variants of αSyn were used in this experiment: *A*, αSynΔ130-140 (Δ130) is a C-terminal truncated version of WT αSyn polypeptide; *B*, αSyn130-140CF (130CF) is a variant where the charged amino acid residues found in the sequence region 130 to 140 were all replaced with Asn residues (Charge-Free); and *C*, αSynY133A/Y136A (YA/YA) is a variant where the two tyrosine residues at positions 133 and 136 were substituted with alanine. The *left most* figures for each variant show fluorescence assays of αSyn fibrillation in the presence and absence of FABP3 and the *black trace* in each panel shows the Thio-T signal of αSynWT in the presence of FABP3. TEM images show the resultant fibrils for each sample at the end of the experimental session (2400 min for *A* and *B*, 3600 min for panel *C*). Scale bars indicate 200 nm. αSyn, α-synuclein; FABP3, fatty acid binding protein 3; Thio-T, thioflavin-T.

Figure 3 and Table 3 show the results of this survey. As shown in Figure 3*A*, we found that the C-terminal sequence region of αSyn was indeed integral to the interaction between this protein and FABP3, as the truncated variant Δ130 became clearly unresponsive to FABP3 addition and retained its tendency to form fibrils. A similar result was also obtained using the truncated αSynΔ120-140 variant. This demonstrates that the C-terminal amino acid residues spanning 130 to 140 of the αSyn amino acid sequence is necessary for αSyn binding to FABP3. With regard to the specific residues within this sequence segment that contributed strongly to FABP3 binding, our results in Figure 3, *B* and *C* show that for each variation of the WT sequence, a significant decrease in the suppressive effects of FABP3 are observable. This suggests that charged amino acid residues and hydrophobic/aromatic Tyr residues both seem to be relevant to the binding of αSyn to FABP3. Most likely, multiple intermolecular interactions involving this segment are involved during binding. However, as observed clearly in Figure 3*A*, the complete excision of this 130 to 140 sequence from αSyn is sufficient to abolish FABP3 binding. It should also be noted here that perturbing a factor, such as charge or hydrophobicity, within this segment did not cause any observable changes in the morphology of the fibrils that were formed by each αSyn variant (compare the TEM images shown in Fig. 3).

**Table 3:** Kinetic values of FABP3 binding to αSyn proteins, αSyn-derived peptides, and arachidonic acid, derived from the experiments shown in Figures 3 and 4

Sample	K_d_ (nM)	*k*_on_ (M^−1^・s^−1^)	*k*_off_ (s^−1^)
FABP3 *versus* αSyn	7.2	6.4 × 10^5^	4.6 × 10^−3^
FABP3 *versus* arachidonic acid	6.8	0.8 × 10^5^	0.6 × 10^−3^
FABP3 *versus* αSynΔ130-140	N.D.	N.D.	N.D.
FABP3 *versus* αSyn130-140CF	8.1	3.0 × 10^5^	2.4 × 10^−3^
FABP3 *versus* αSynY133A/Y136A	61.7	0.9 × 10^5^	5.5 × 10^−3^
FABP3 *versus* αSynP_2-10_	N.D.	N.D.	N.D.
FABP3 *versus* αSynP_73-96_	N.D.	N.D.	N.D.
FABP3 *versus* αSynP_130-140_	6.1	2.0 × 10^5^	1.2 × 10^−3^
FABP3 F16S *versus* αSyn	N.D.	N.D.	N.D.
FABP3 F16S *versus* αSynP_130-140_	N.D.	N.D.	N.D.
FABP3 F16S *versus* arachidonic acid	2090	0.02 × 10^5^	4.7 × 10^−3^
FABP3 *versus* αSynP_130-140_Y133F	2.0	7.5 × 10^5^	1.5 × 10^−3^
FABP3 *versus* αSynP_130-140_Y136F	7.7	4.8 × 10^5^	3.7 × 10^−3^
FABP3 *versus* αSynP_130-140_Y136W	6.3	3.8 × 10^5^	2.4 × 10^−3^
FABP3 *versus* αSynP_130-140_ Y133F/Y136F	2.1	5.4 × 10^5^	1.1 × 10^−3^
FABP3 *versus* αSynP_130-140_ Y133F/Y136W	6.1	3.2 × 10^5^	1.9 × 10^−3^
FABP3 *versus* αSynP_130-140_Y133W/Y136W	6.2	2.4 × 10^5^	1.5 × 10^−3^

αSyn, α-synuclein; FABP3, fatty acid binding protein 3; N.D., not determined.

**Figure 4:**
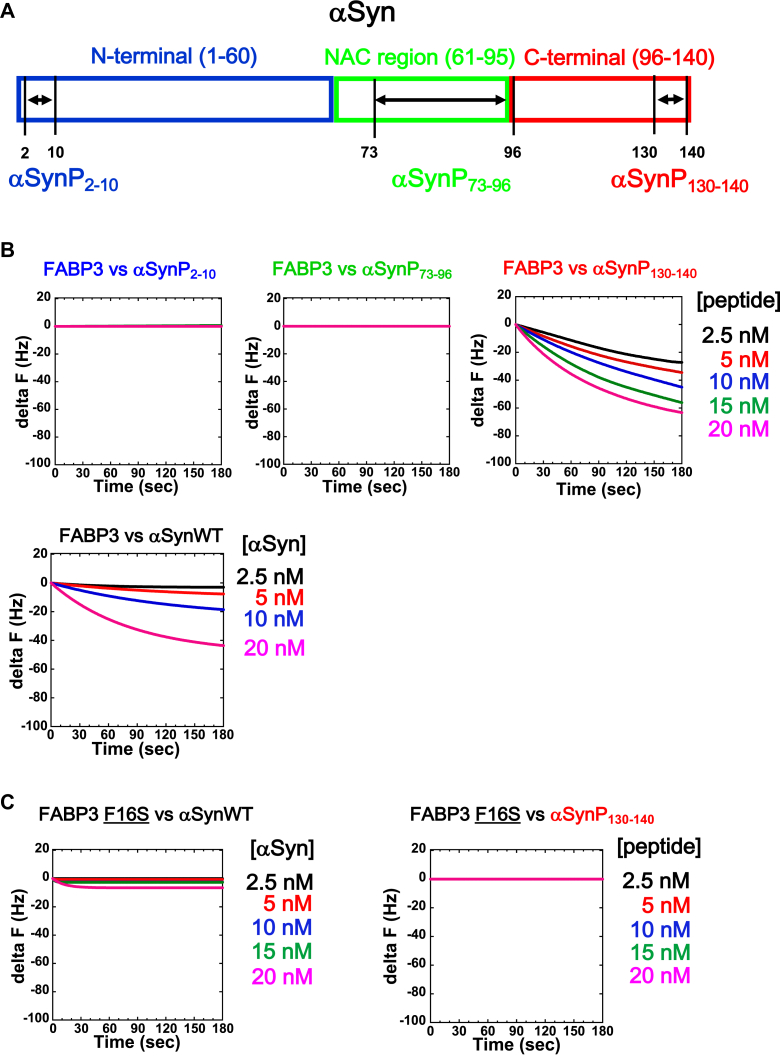
**Quantitation of the affinity between FABP3 and αSyn-derived oligopeptides.***A*, location of each peptide segment (αSynP) mapped to a sequence schematic of αSynWT. *B*, QCM analysis of each peptide to immobilized FABP3. The concentration of peptide added to the sensor cell for each trace is shown to the *right* of the figure. A reference analysis for binding of intact αSyn protein to immobilized FABP3 is also shown (“*FABP3 versus αSynWT*”). *C*, QCM analysis of αSynWT or αSynP130-140 to immobilized FABP3 F16S mutant. Binding of αSynP130-140 is largely abolished in the FABP3 F16S mutant. αSyn, α-synuclein; FABP3, fatty acid binding protein 3; QCM, quartz crystal microbalance.

Having determined the importance of the 130 to 140 sequence region in αSyn for FABP3 binding, we next set out to probe this sequence segment in more detail, using chemically synthesized peptides. In Figure 4*A*, we highlight this sequence segment along with two other sequences found within the αSyn sequence that we use for comparison: the N-terminal sequence region (αSynP2-10; nine residues) and the segment 73 to 96 in the NAC region of the sequence (αSynP73-96; 24 residues). The αSynP73-96 peptide is of special interest because this sequence has been implicated in previous studies as an important component of the nucleation core of αSyn fibrils (31, 32). In Figure 4*B*, we show the results of a quartz crystal microbalance (QCM) binding assay that directly measures binding between immobilized FABP3 and these three model peptides, along with binding assays using the intact WT αSyn protein (αSynWT). The kinetic constants derived from these assays are shown in Table 3, and the sequence of the peptides are shown in Table 4. As shown in Figure 4*B*, we determined from QCM assays that the peptide αSynP130-140 specifically bound to immobilized FABP3, whereas the other two peptides αSynP2-10 and αSynP73-96 did not bind under the conditions we tested. The K_d_ for αSynP130-140, estimated to be 6.1 nM, was comparable to the affinity constant of FABP3 for its cellular component, arachidonic acid (6.8 nM) and for intact αSyn protein (7.2 nM). Judging from the binding constants summarized in Table 3, αSynΔ130-140 was incapable of binding to FABP3 under the conditions we applied. These findings highlighted again the relative importance of the amino acid 130 to 140 sequence region for the binding to FABP3 of αSyn.

**Table 4:** Sequences and molecular weights of αSyn-derived peptides used in the experiments shown in Figures 4 and 6

Sample	Molecular weight	Amino acid sequence
αSynP_2-10_	1095.3	^2^DVFMKGLSKA^10^
αSynP_73-96_	2232.6	^73^GVTAVAQKTVEGAGSIAAATGFVK^96^
αSynP_130-140_	1329.2	^130^EEGYQDYEPEA^140^
αSynP_130-140_ Y133F	1314.2	^130^EEGFQDYEPEA^140^
αSynP_130-140_ Y136F	1313.4	^130^EEGYQDFEPEA^140^
αSynP_130-140_ Y136W	1352.9	^130^EEGYQDWEPEA^140^
αSynP_130-140_ Y133F/Y136F	1297.2	^130^EEGFQDFEPEA^140^
αSynP_130-140_ Y133F/Y136W	1336.6	^130^EEGFQDWEPEA^140^
αSynP_130-140_ Y133W/Y136W	1375.2	^130^EEGWQDWEPEA^140^

αSyn, α-synuclein.

Next, to demonstrate that binding of αSynP130-140 to FABP3 did in fact involve a specific binding site, we mutated the Phe16 site of FABP3 to Ser and probed the mutant protein’s ability to bind to αSyn and its derivative peptides. Phe16 is located within the fatty acid binding pocket of FABP3 (33) and has previously been demonstrated to be an important residue for the binding of arachidonic acid by FABP3 (26), an integral characteristic for its cellular activity. As shown in Figure 4*C* and Table 3, we found that the mutant FABP3 F16S was now unable to bind the peptide αSynP130-140 and αSynWT. Binding affinities of FABP3 F16S for arachidonic acid decreased significantly (K_d_ = 2090 nM) (Table 3), suggesting that the binding site for arachidonic acid and αSyn on FABP3 may overlap and include the fatty acid binding pocket. A preliminary molecular simulation analysis of αSynP130-140 binding to the fatty acid binding site of FABP3 was performed using AutoDock Vina (34) (http://vina.scripps.edu/), and a plausible complex structure was derived of a state with reasonable energy minima (−7.3 kcal/mol). Of note, in this putative structure Tyr133 of αSynP130-140 was proposed to interact with Phe16 of FABP3. These analyses also suggested the importance of the C-terminal region of αSyn to the binding of FABP3, to form the αSyn–FABP3 complex.

**Figure 5:**
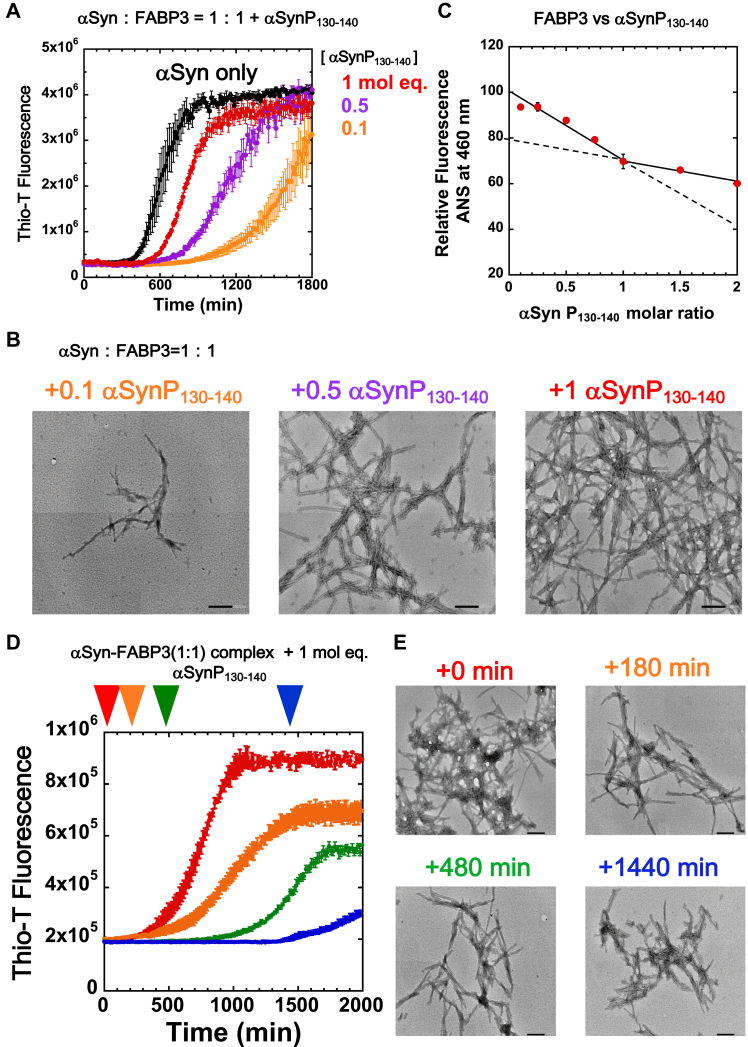
**Addition of αSynP130-140 inhibits formation of the αSyn–FABP3 complex and allows the formation of αSyn fibrils.***A*, addition of increasing concentrations of αSynP130-140 abolishes the suppressive effects of FABP3 on αSyn fibrillation. The molar equivalents of αSynP130-140 added to samples containing 69 μM αSyn and 69 μM FABP3 is denoted to the *right of the panel*. *B*, TEM images of aliquots taken from fluorescence assay samples shown in panel (*A*). Scale bars indicate 200 nm. *C*, changes in ANS fluorescence for FABP3 samples in the presence of increasing concentrations of αSynP130-140, showing that the decrease in fluorescence at 460 nm plateaus at roughly a 1:1 molar ratio of FABP3 to peptide. *D*, effects of delayed addition of 69 μM αSynP130-140 to samples containing 69 μM αSyn and 69 μM FABP3 mixtures. *Red*, *orange*, *green*, and *blue arrows* denote delayed addition of αSynP130-140 after an initial incubation of 0, 180, 480, and 1440 min, respectively. *E*, TEM images of samples shown in panel (*D*) after completion of the assay (2000 min). Scale bars indicate 200 nm. αSyn, α-synuclein; αSynP, synthetic peptide derived from α-synuclein; ANS, 1-anilino-8-napthalene sulfonate; FABP3, fatty acid binding protein 3; TEM, transmission electron microscopy.

Because our QCM experiments suggested that the αSynP130-140 peptide (K_d_ = 6.1 nM) and intact αSyn (K_d_ = 7.2 nM) were binding to FABP3 with roughly similar affinities (Table 3), we tested if the peptide would be able to interfere with the binding of these two proteins and elicit an effect on the formation of αSyn fibrils in the presence of FABP3. As shown in Figure 5, *A* and *B*, this was indeed the case, and the addition of increasing amounts of αSynP130-140 served to effectively prevent the binding of FABP3 to αSyn, which resulted in αSyn recovering its tendency to form fibrils under our experimental conditions. Both fluorescence and TEM imagery were consistent with a mechanism in which αSynP130-140 bound to FABP3 in place of intact αSyn, which allowed the latter protein to form detectable fibrils. Furthermore, as shown in Figure 5*C*, binding assays of αSynP130-140 for FABP3 using ANS fluorescence showed that the decrease in fluorescence at 460 nm saturates at roughly a 1:1 molar ratio of FABP3 to peptide, suggesting 1:1 binding stoichiometry.

In Figure 5, *D* and *E*, we show the effects of delayed αSynP130-140 addition to αSyn–FABP3 mixtures to highlight an interesting effect of the peptide on αSyn–FABP3 interaction. As seen in the figure, for each case tested, delayed addition of αSynP130-140 to premixed samples of αSyn–FABP3 resulted in the formation of Thio-T–sensitive fibril αSyn species after a suitable lag time related to the delay. Of special note is the sample where peptide was added to αSyn–FABP3 after 1440 min. As shown in Figure 5*A*, under the conditions we studied, formation of Thio-T-sensitive αSyn fibrils is typically complete after an incubation of ∼800 min. The *blue* trace in Figure 5*D*, therefore, represents a demonstration that FABP3 binding is capable of suppressing this fibrillation for the duration of this time frame. Following this, the finding that addition of αSynP130-140 causes a resumption of Thio-T sensitive fibrillation may be taken as evidence that αSynP130-140 is able to disrupt the αSyn–FABP3 interaction that is suppressing fibrillation. In other words, the nature of the interactions between FABP3 and αSynP130-140 is such that the peptide is capable of dislodging prebound Syn from the fatty acid binding site of FABP3.

### Synthesized C-terminal peptides derived from αSyn modulate αSyn–FABP3 binding

**Figure 6:**
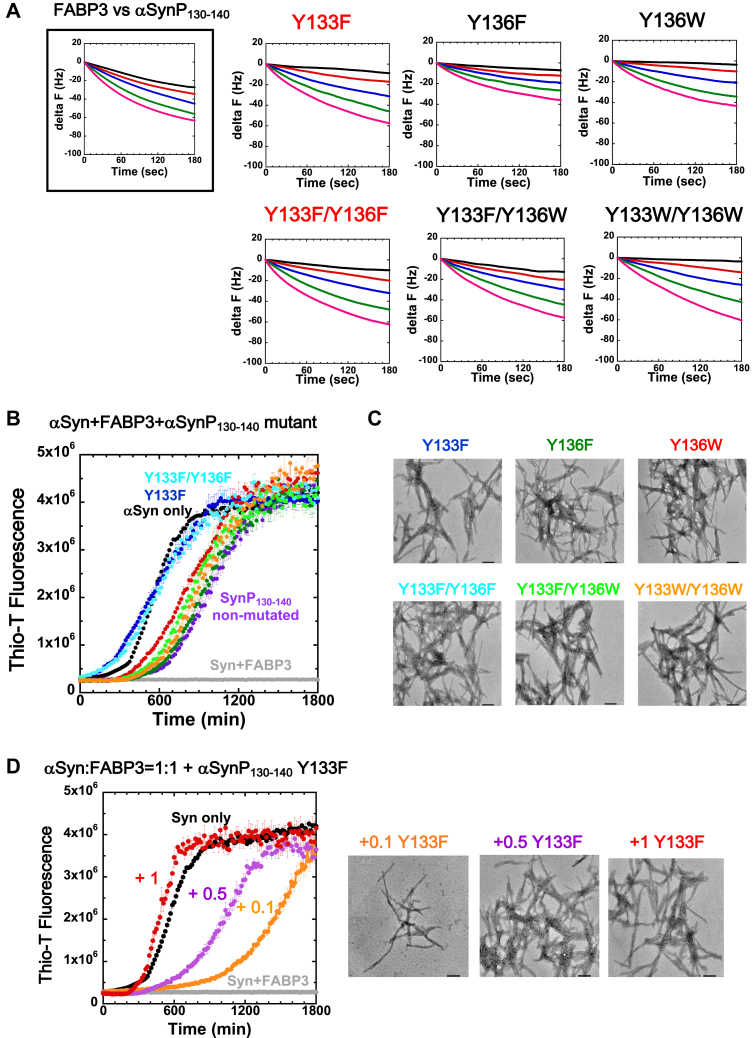
**Interaction affinity analysis of Tyr-to-*aromatic* residue substituted αSynP130-140 peptides with FABP3 and evaluation of the effects of peptide binding on the αSyn fibril formation process.***A*, QCM analysis of interaction between FABP3 and Tyr substituted αSynP130-140 peptides. For comparison, analysis figure of “*FABP3 versus αSynP130-140*” was indicated at *top left* by reusing from panel (*B*) of Figure 4. *B*, effects of αSynP130-140 peptides with Tyr residue substitution and FABP3 on αSyn fibril formation. αSyn only (*black*), αSyn+FABP3 (*gray*), αSyn+FABP3+αSynP130-140 (nonmutated) (*purple*), αSyn+FABP3+Y133F (*blue*), αSyn+FABP3+Y136F (*green*), αSyn+FABP3+Y136W (*red*), αSyn+FABP3+Y133F/Y136F (*light blue*), αSyn+FABP3+Y133F/Y136W (*light green*), and αSyn+FABP3+Y133W/Y136W (*orange*). *C*, TEM images of amyloid fibrils formed at 1800 min in panel (*B*). Scale bars indicate 200 nm. *D*, effects of adding various molar equivalents of Y133F to 69 μM FABP3 and 69 μM αSyn mixtures on αSyn fibril formation, with accompanying TEM images taken at 1800 min. αSyn only (69 μM) (*black*), αSyn:FABP3 = 1:1 (69 μM:69 μM) (*gray*), αSyn:FABP3:Y133F = 1:1:0.1 (69 μM:69 μM:6.9 μM) (*orange*), αSyn:FABP3:Y133F = 1:1:0.5 (69 μM:69 μM:34.5 μM) (*magenta*), and αSyn:FABP3:Y133F = 1:1:1 (69 μM:69 μM:69 μM) (*red*). Scale bars indicate 200 nm. αSyn, α-synuclein; αSynP, synthetic peptide derived from α-synuclein; FABP3, fatty acid binding protein 3; QCM, quartz crystal microbalance; TEM, transmission electron microscopy.

Previous studies (26, 35, 36) have suggested that an interaction between αSyn and FABP3 may be a significant factor in the onset of cell morbidity and neurological degradation in the case of PD. In this context, our experimental results that show that a synthetic peptide may disturb this interaction would also be of interest from a clinical standpoint and may possibly provide a means to prevent or treat this degeneration. With this in mind, we set out to see if the interactions between FABP3 and αSynP130-140 may be tuned toward higher affinity, through substitution of certain residues in the peptide sequence. As shown in Figure 6, we focused on two aromatic side chains, Tyr133 and Tyr136, and substituted them with other aromatic amino acids to gauge the response of FABP3-peptide interaction to side chain substitution. The importance of these two Tyr residues were highlighted in the binding experiments performed with αSynY133A/Y136A (Fig. 3*C* and Table 3). As shown in Figure 6*A*, we found that substituting Tyr133 with Phe resulted in a three-fold increase in affinity in terms of K_d_ between FABP3 and αSynP130-140 (see also Table 3). However, this increase was not enhanced by an additional substitution of Tyr136 to Phe; interestingly, a simple substitution of only Tyr136 to Phe did not cause any increase in affinity. We also found that the increase in peptide affinity toward FABP3 could only be realized with the Tyr133 to Phe substitution, as a Tyr-to-Trp substitution at the 133 and/or 136 sites did not elicit an increase in binding affinity (Table 3). This finding may be rationalized from the AutoDock simulation results described above which highlighted the importance of the interaction between Tyr133 (αSynP130-140) and Phe16 (FABP3).

The increased affinity of the αSynP130-140_Y133F and αSynP130-140_Y133F/Y136F peptides was also reflected in αSyn fibrillation assays, as shown in Figure 6, *B* and *C*. Whereas all of the peptides surveyed in this experiment were successful in offsetting the suppressive effects of FABP3 on αSyn fibrillation (compare the *gray* “Syn+FABP3” trace in Fig. 6*B* to the *colored* traces), we saw that the αSynP130-140_Y133F and αSynP130-140_Y133F/Y136F peptides were significantly more successful in neutralizing the effects of FABP3 binding, to a degree that the fibrillation reaction resembled the trace seen in the absence of FABP3 (Fig. 6*B*, *black* trace). Also, as shown in Figure 6*D*, an equimolar concentration of αSynP130-140_Y133F was sufficient in these experiments to completely suppress the effects of FABP3 addition, and substoichiometric concentrations of peptide resulted in a concentration-dependent suppression of the FABP3-induced fibril suppression effect. Our results suggest that there may be merits to testing this modified peptide for protective effects on cellular morbidity in model systems for neurodegenerative disorders involving the intracellular aggregation and deposition of αSyn.

### Consequences of αSyn–FABP3 complex to cellular viabilities

**Figure 7:**
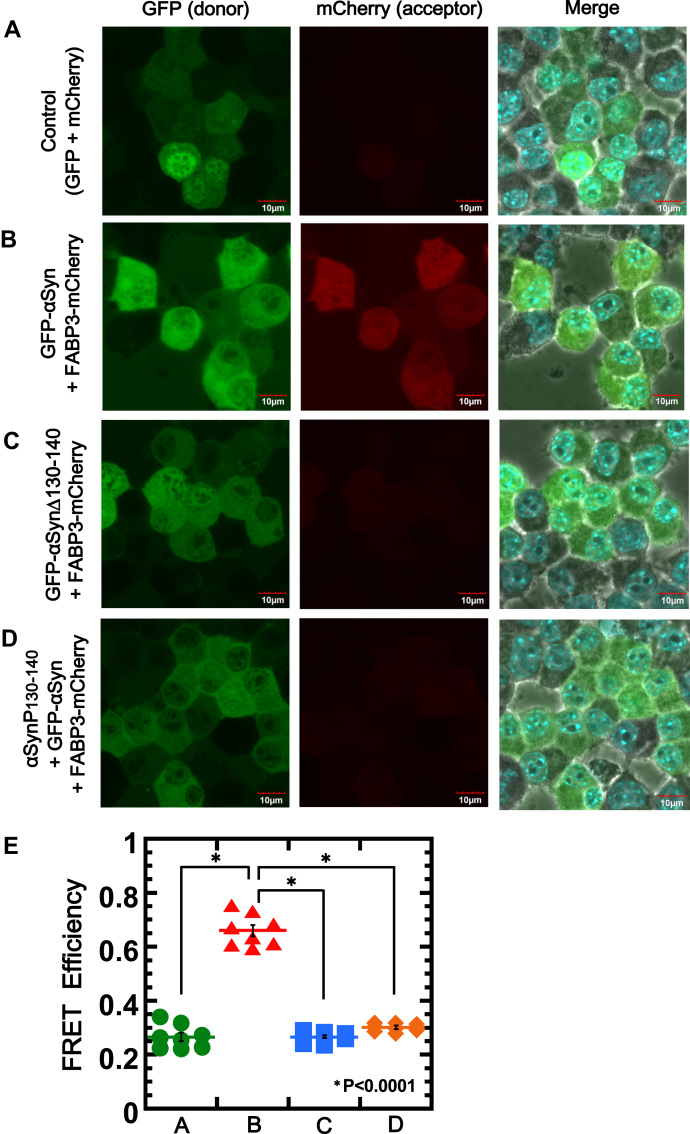
**Evaluation of interactions between αSyn and FABP3 in cells using FRET.** FRET experiments were performed using N2a cells co-expressing GFP-αSyn and FABP3-mCherry. FRET from GFP to mCherry was measured (GFP Ex: 489 nm/Em: 510 nm, mCherry Em: 610 nm). *A*, FRET measurements of control N2a cells expressing GFP and mCherry. *B*, FRET measurement of N2a cells expressing GFP-αSyn and FABP3-mCherry. *C*, FRET measurement of N2a cells expressing GFP-αSynΔ130-140 and FABP3-mCherry. *D*, FRET measurement of N2a cells expressing GFP-αSyn and FABP3-mCherry in the presence of 50 μM αSynP130-140. *E*, FRET efficiencies of A, B, C, and D were calculated from (total fluorescence intensity of mCherry/total fluorescence intensity of GFP) of each cell using Image-J software. ∗*p* < 0.0001. αSyn, α-synuclein; αSynP, synthetic peptide derived from α-synuclein; FABP3, fatty acid binding protein 3; FRET, fluorescence resonance energy transfer; GFP, green fluorescent protein; N2a, Neuro2A.

To demonstrate the presence of a direct interaction between αSyn and FABP3 in cells, we performed FRET experiments on Neuro2A (N2a) cells simultaneously expressing green fluorescent protein (GFP)-αSyn and FABP3-mCherry (37). Expression of both GFP-αSyn and FABP3-mCherry was confirmed, and FRET efficiencies between these two proteins were evaluated under a range of conditions. As shown in Figure 7, whereas FRET signals from GFP to mCherry were not observed for control cells co-expressing GFP and mCherry (Fig. 7*A*), a significant FRET signal was observed in cells co-expressing the two fusion constructs GFP-αSyn and FABP3-mCherry (Fig. 7*B*, *center*). This signal was not observed when GFP–αSynΔ130-140 was expressed instead of GFP-αSynWT (Fig. 7*C*, *center*), and more significantly, the signal was abolished upon the addition of αSynP130-140 peptide to the culture (Fig. 7*D*, *center*). The estimated FRET signal efficiencies (Fig. 7*E*) (total fluorescence intensity of mCherry/total fluorescence intensity of GFP) from each cell also demonstrated clearly that the FABP3–αSyn complex formed *via* the C-terminal region (130–140) of αSyn was detectable even in cells, complementing the *in vitro* experiments.

**Figure 8:**
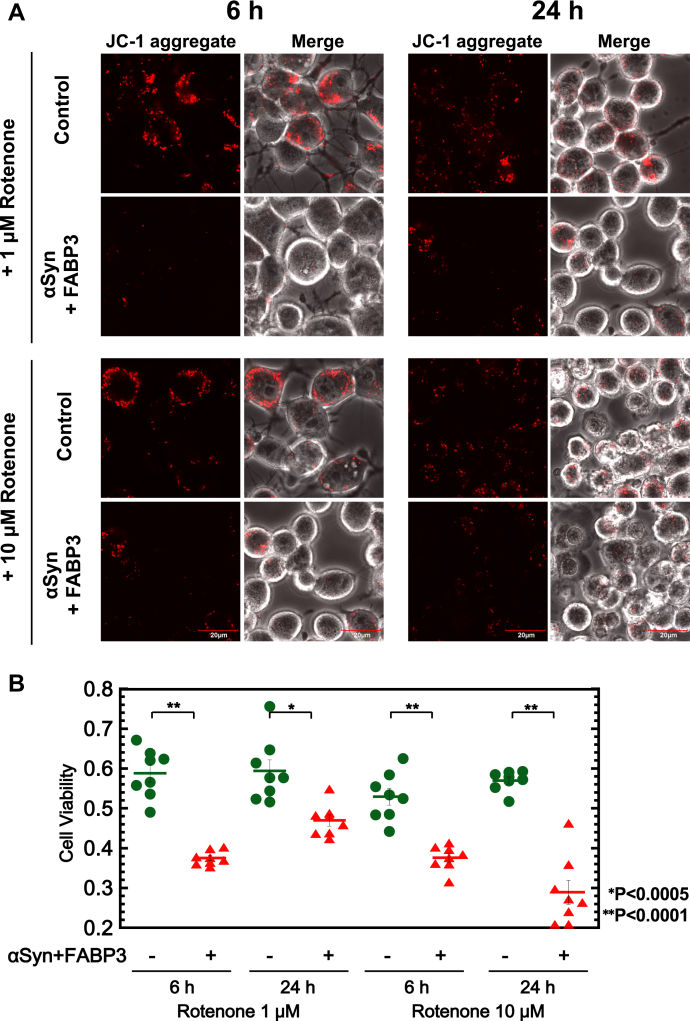
**Measurements of mitochondrial membrane potential in N2a cells co-expressing αSyn and FABP3, by monitoring JC-1 fluorescence under various conditions.***A*, N2a cells were incubated in the presence of 1 μM or 10 μM rotenone for either 6 h and 24 h. *B*, cell viabilities were calculated from [total fluorescence intensity of JC-1 aggregate/total fluorescence intensity of (JC-1 aggregate + JC-1 monomer)] of each cell, as quantitated using Image-J software. ∗*p* < 0.0005, ∗∗*p* < 0.0001. αSyn, α-synuclein; FABP3, fatty acid binding protein 3; JC-1, 5, 5’, 6, 6’-tetrachloro-1, 1’, 3, 3’-tetraethylbenzimidazolylcarbocyanine iodide; N2a, Neuro2A.

**Figure 9:**
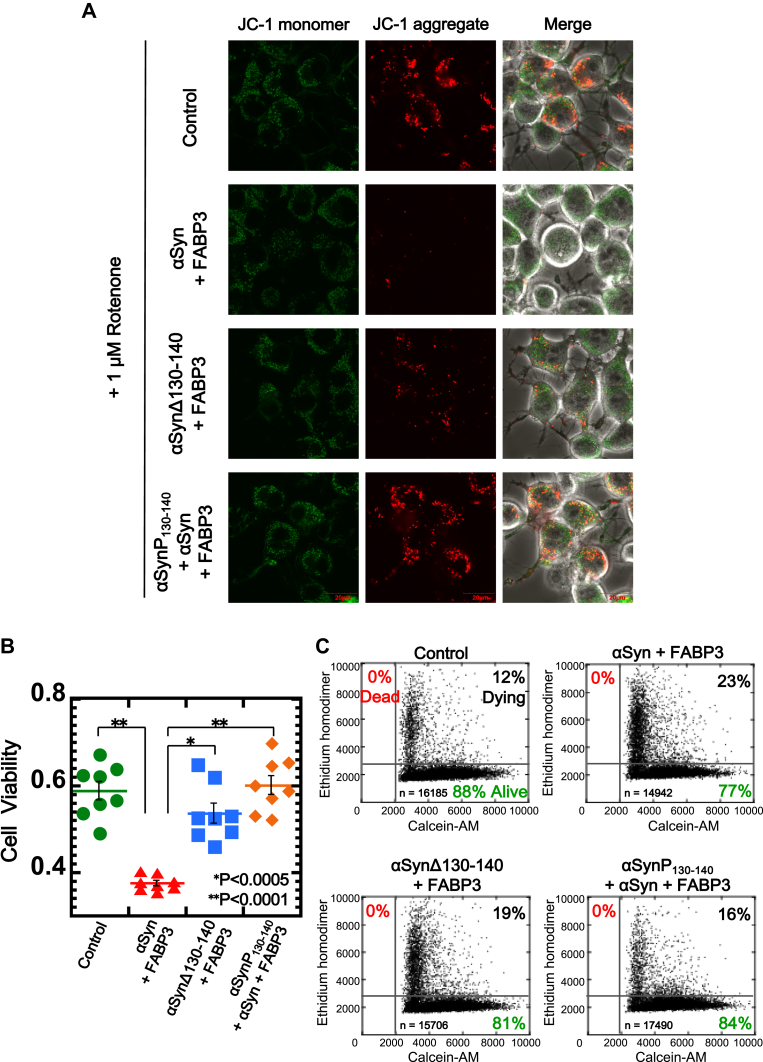
**Cytotoxicity measurements of αSyn–FABP3 complex in N2a cells by using a JC-1 probe and Tali cytometer.***A*, measurements of mitochondrial membrane potential by staining with JC-1 of αSyn and FABP3 expressing N2a cells incubated in the presence of various factors and 1 μM rotenone for 6 h. JC-1 aggregate fluorescence image in Control was reused from the control fluorescence image in panel (*A*) of Fig. 8. *B*, cell viabilities were calculated from [total fluorescence intensity of JC-1 aggregate/total fluorescence intensity of (JC-1 aggregate + JC-1 monomer)] of each cell using Image-J software. ∗*p* < 0.0005, ∗∗*p* < 0.0001. *C*, cell viabilities of N2a cells co-expressing αSyn and FABP3 that were incubated for 24 h in the presence of various factors and 30 μM 6-OHDA, as measured using the Tali Cytometer. Cells co-stained with Calcein-AM and EthD-1 represent dying cells. 6-OHDA, 6-hydroxydopamine; αSyn, α-synuclein; EthD-1, ethidium homodimer; FABP3, fatty acid binding protein 3; JC-1, 5, 5’, 6, 6’-tetrachloro-1, 1’, 3, 3’-tetraethylbenzimidazolylcarbocyanine iodide; N2a, Neuro2A.

Additionally, the viabilities of N2a cells co-expressing αSyn and FABP3 were also evaluated. Cytotoxicity was assayed by using two different methods: a 5, 5’, 6, 6’-tetrachloro-1, 1’, 3, 3’-tetraethylbenzimidazolylcarbocyanine iodide (JC-1) fluorescent probe (38) to monitor mitochondrial integrity and a Tali cytometer that monitors cell membrane integrity (39). First, as shown in Figure 8*A*, we monitored cell viabilities using the JC-1 probe under various culture conditions (in the presence of 1 and 10 μM rotenone (40) for 6 and 24 h). The results showed that under all conditions tested, formation of the αSyn–FABP3 complex caused significant damage to the mitochondrial membrane that was detectable by changes in JC-1 fluorescence (Fig. 8*B*). Next, we examined the effects of substituting αSynΔ130-140 for αSynWT and adding αSynP130-140 peptide to the cultures with FABP3 and αSyn co-expression. Figure 9, *A* and *B* suggested that damage to the mitochondrial membrane was not observed in cells co-expressing αSynΔ130-140 and FABP3 (in the presence of 1 μM rotenone), and the effects seen for FABP3 and αSynWT co-expression were neutralized upon addition of αSynP130-140 peptide to the culture. As shown in Figure 9*C*, changes in cell viability similar to those detected using JC-1 were also observed in Tali measurements in the presence of 30 μM 6-hydroxydopamine (41), monitored through changes in the fluorescence of Calcein-AM/ethidium homodimer (42). For cells co-expressing αSyn and FABP3, a significant increase [from 12% (control) to 23%] in cells co-stained with Calcein-AM and ethidium homodimer was observed, reflecting an increase in dying cells (43). The number of dying cells did not increase to the same degree in cultures of cells expressing αSynΔ130-140 instead of αSynWT (19%) or in cultures grown in the additional presence of αSynP130-140 peptide (16%). These results demonstrated that the formation of the αSyn–FABP3 complex in cells results in an increase in cytotoxicity that is sensitive to perturbations that focus on the C-terminal region of αSyn.

## Discussion

The deposition of Lewy bodies, composed of αSyn aggregates, in neurons is considered to be an important event in the progression of PD (44, 45, 46). Many studies have demonstrated that a multitude of cellular factors contribute to this intracellular deposition of αSyn (47, 48). Recently it has been inferred that the fatty acid binding protein FABP3 is also implicated in this process, through *in vivo* experiments that demonstrate a specific requirement of FABP3 for the deposition of αSyn fibrils (35, 36). In this report, we offer both *in vitro* and *in vivo* evidence that confirms the existence of a relationship between these two cellular proteins and provide details regarding its molecular mechanism.

The presence of FABP3 in fibrillization experiments of αSyn served to strongly suppress the formation of Thio-T sensitive αSyn fibrils (Fig. 1). This suppressive effect was traced to the formation of a 1:1 binary complex of αSyn and FABP3 which initially prevented the formation of higher molecular fibrillar aggregates (Fig. 2). This effect may be attributed to an occlusion of the NAC region of αSyn by FABP3 that would inhibit the formation of β-structure αSyn amyloid nuclei. Upon extended incubation, however, the αSyn–FABP3 binary complex evolved into a different form that was structurally distinct from the fibrillar aggregates formed by αSyn alone [such as (αSyn-FABP3)n oligomeric species], in molecular size and surface hydrophobicity. A curious similarity however was observed between the intermediate aggregate species formed in the absence and presence of FABP3; both molecular forms proved to be toxic, with the strongest toxicity observed at about 6 h after initiation of the fibrillation reaction in the 3-(4,5-dimethylthiazol-2-yl)-5-(3-carboxymethoxyphenyl)-2-(4-sulfophenyl)-2H-tetrazolium (MTS) assay. This toxicity was gradually lost as the intermediates matured to form Thio-T sensitive fibrils in samples containing αSyn only. In samples containing both αSyn and FABP3, extended incubation resulted in the formation of an alternative soluble aggregate distinct from the fibrillar species [(αSyn-FABP3)n oligomer]. Based upon our results, our present hypothesis proposes that the interaction with FABP3 serves to suppress amyloid fibril formation of αSyn by forming of αSyn–FABP3 complex that gradually converts to oligomers that are cytotoxic. The differences in the high molecular aggregates that αSyn forms in the absence and presence of FABP3 may be related to the experimental finding that in cells lacking FABP3, researchers were unable to detect any significant intracellular deposits of insoluble αSyn. We also would like to note that this difference also translated into a difference in cellular viability, which suggests a pathologic role for this interaction *in vivo* (36, 49).

Although this idea of toxic FABP3–αSyn oligomers forming intracellularly would explain our experimental results sufficiently, extension of this idea brings about a paradox; specifically, that if this idea were to be true, there should be a certain amount of toxic FABP3–αSyn complexes that form under normal cellular conditions in WT cells, to the detriment of cellular viability. Although our data at present do not prove or disprove this possibility, one explanation for our results may be that in WT cells, the amount of FABP3 that is normally expressed may be too low to allow apo (non lipid-bound) FABP3 to accumulate to levels that would be detectable in our assays (unlike in our FABP3-overproducing cells, where the amount of lipid would limit the amount of holo-FABP3). This difference in expression may be translated to differences in cellular oligomer levels and thereby, detectable cellular toxicity.

To determine the specific sites and mechanism of the binding interaction between αSyn and FABP3, we utilized QCM to monitor directly the binding between FABP3 and αSyn full-length protein or derived synthetic peptides, in combination with mutational analysis (Figs. 3 and 4). In previous studies, the C-terminal segment of the αSyn polypeptide has been highlighted as a vital region involved in αSyn oligomerization and fibrillation, where mutations to the sequence cause changes in the fibril kinetics, morphology, and sensitivity to changes in the surrounding environment (28, 29, 30). The C-terminal region of αSyn has also been implicated in interactions between tau protein and its evolution into toxic molecular species (50). In light of these previous findings, we elected to focus on this section of the αSyn amino acid sequence for insights regarding FABP3 recognition and binding.

Our experiments demonstrated that the C-terminal region of αSyn was indeed integral to the recognition and binding to FABP3 both *in vitro* and *in vivo* (Figure 5, Figure 6, Figure 7, and Table 3). Interestingly, identification of this region may be utilized to control the fate of αSyn oligomerization and fibrillation to a certain extent. Our evidence for this assertion is threefold: (1) Deletion of the last 11 residues of αSyn abolishes the ability of αSyn to bind to FABP3, (2) a synthetic peptide corresponding to this deleted sequence is capable of effectively disrupting the αSyn–FABP3 complex through competitive binding, and (3) mutation of a specific phenylalanine residue (F16) (51, 52) in FABP3 that is implicated in the binding of PUFA to FABP3 was also sufficient to abolish binding to αSyn. This last finding holds various implications for the numerous functional roles and dynamic interactions between fatty acids, FABP3, and αSyn that may be relevant to cellular viability and neuropathological progression. As a *de-facto* competitive inhibitor to fatty acids toward FABP3, the presence of αSyn in excessive amounts could conceivably perturb the import of these molecules into cells that require them for maintaining viability. In this context, it should be noted that recently, an interesting study focusing on a relationship between a decrease in the population of short-chain fatty acid–producing gut flora and PD pathology was reported (53). Additionally, the affinity between FABP3 and αSyn may act as a vector by which various types of αSyn may be transported into cells, as was recently demonstrated for tau protein by LRP1 (54). These intriguing aspects regarding the *in vivo* aspects and consequences of FABP3–αSyn binding are being actively probed (35). Recent studies toward this objective have revealed that FABP3 is indeed relevant to αSyn import into mammalian cells and also that the interaction between the C-terminal region of αSyn and FABP3 is critical to this import (55, 56).

Finally, we consider the significance of developing an ability to destabilize the αSyn–FABP3 complex in cells. From the *in vitro* experiments (Fig. 1, *A* and *B*), αSyn–FABP3 complex formation seemed at first glance to be potentially beneficial, judging from the significant suppressive effects on αSyn fibrillation. However, as shown in Figures 8 and 9, we found that the formation of αSyn–FABP3 complex in cells clearly resulted in a disruption in mitochondrial membrane potential and a concomitant decrease in cell viability by forming (αSyn-FABP3)n oligomeric species. From these results, we propose that instead of maintaining and/or stabilizing the αSyn–FABP3 complex, it would be more beneficial to the cells to add a ligand, such as αSynP130-140_Y133F, that would release αSyn from FABP3 and allow αSyn to freely form mature and nontoxic amyloid fibrils (as shown in Fig. 6*D*). It is reported in multiple studies that mature amyloid fibrils of αSyn are not toxic and instead intermediate oligomers seen during fibril formation are most cytotoxic (7, 9, 10). Therefore, conditions where the toxic intermediates are stabilized (*i. e.*, due to binding to FABP3) would result in a net negative effect on cellular viability and should be avoided.

**Figure 10:**
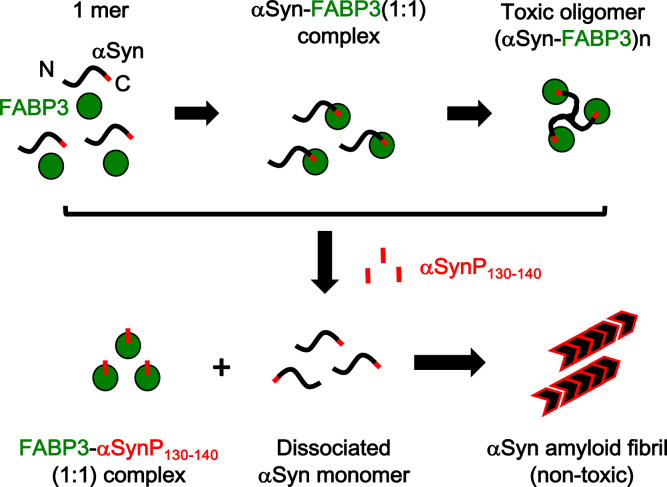
**Schematic model of αSyn fibrillation in the presence of FABP3 and αSynP130-140.** αSyn initially assumes a monomeric random structure in solution. FABP3 recognizes the C-terminal peptide region of αSyn (denoted in *red*), binds to it, and forms a soluble αSyn–FABP3 complex at a 1:1 molar ratio. By forming this complex, fibrillation of αSyn is suppressed. This αSyn–FABP3 complex undergoes changes in size and structure over time to an oligomeric molecular species [(αSyn-FABP3)n] that displays cytotoxicity. When the αSynP130-140 peptide is present in these αSyn–FABP3 complexes, αSynP130-140 displaces full length αSyn to be dissociated, which subsequently resumes the formation of mature amyloid fibrils that are not toxic. αSyn, α-synuclein; αSynP, synthetic peptide derived from α-synuclein; FABP3, fatty acid binding protein 3.

Figure 10 is a schematic that summarizes the results obtained in our experiments that elucidate the interactions between αSyn and FABP3. Highlights of our results include detection and characterization of a binding relationship between αSyn and FABP3 that results in production of toxic oligomeric species that are relevant to the progression of synucleinopathies. Also of note is the fact that we were successful in producing an αSyn-derived peptide with a high affinity toward FABP3 that could conceivably be used as an inhibitor of αSyn–FABP3 binding (57, 58). Owing to its relatively small size, such peptides are an attractive candidate for clinical applications aimed toward control and treatment of diseases that involve αSyn aggregation, and conceivably, fatty acid transport as well.

## Experimental procedures

### Materials

Peptides derived from the αSyn amino acid sequence (αSynP2-10; DVFMKGLSKA, SynP73-96; GVTAVAQKTVEGAGSIAAATGFVK, αSynP130-140; EEGYQDYEPEA, and Tyr-to-Phe/Trp mutated peptides of αSynP130-140) were synthesized by SCRUM Inc or BEX Co Ltd. Peptides were dissolved in 50 mM Tris-HCl, pH 7.4 at 37 °C or 25 °C before use.

pCAG-GFP and mCherry2-N1 genes were a gift from Connie Cepko (Addgene plasmid # 11150; http://n2t.net/addgene:11150; RRID:Addgene_11150) and from Michael Davidson (Addgene plasmid # 54517; http://n2t.net/addgene:54517; RRID:Addgene_54517), respectively. The plasmid pCAG-GFP-αSyn was previously constructed in our laboratory (59). pCAG-FABP3-mCherry was prepared by substituting the GFP ORF of pCAG-GFP with a synthetic FABP3-mCherry gene (Integrated DNA Technologies). pCAG-αSyn and pCAG-FABP3 were prepared from pCAG-GFP-αSyn and pCAG-FABP3-mCherry by excising the GFP and mCherry sequences, respectively.

### Preparation of protein

FABP3 and FABP3 with N-terminal His6 tag (His6-FABP3) were each expressed in *E. coli* BLR(DE3) cells using a pET28a-derived vector containing coding genes synthesized by Invitrogen GeneArt gene synthesis. The genes were inserted into pET28a using either the restriction sites *Nco*I and *Xho*I (FABP3) or *Nde*I and *Xho*I (His6-FABP3). Expression vectors were confirmed by sequencing before use in expressing proteins. Purification of FABP3 and His6-FABP3 were performed according to published protocols. Briefly, cells grown to OD_600_ = 0.6∼0.8 were induced by the addition of 1 mM isopropyl-β-D-thiogalactopyranoside (IPTG, Wako) to the medium, followed by cultivation for 15 h at 37 °C. Cells were recovered by centrifugation (8000 rpm, 20 min, 4 °C), resuspended in 50 mM Tris-HCl, pH 7.4 at 4 °C in a 10:1 (w:v) ratio of cells to buffer, and the cell suspension was disrupted sonically using a Branson sonifier 450. Supernatant fractions were recovered using centrifugation (14,500 rpm, 20 min, 4 °C), and contaminating nucleic acid was removed from this clarified supernatant by the gradual addition of 2.5% (w/v) streptomycin sulfate. After a 30 min incubation at room temperature, the pellet fraction containing nucleic acid was removed by centrifugation, and the supernatant was brought to 80% saturation by adding solid ammonium sulfate. After recovery of the resultant protein precipitate using centrifugation, the protein fractions containing FABP3 or His6-FABP3 were resolubilized in 50 mM Tris-HCl, pH 7.4 at 25 °C and applied to purification columns attached to an AKTA-FPLC system (GE Healthcare) operating at room temperature. A Resource-Q anion exchange column (GE Healthcare) was used to purify FABP3 during this step, and a His-trap HP nickel-affinity column (GE Healthcare) was used to purify His6-FABP3. After development of the column, fractions containing the desired protein were pooled, and a 0.4-fold volume of diisopropylether:*n*-butanol (3:2) solution was added. The mixture was allowed to stand at room temperature with gentle agitation (30 rpm on a reciprocal shaker) for three cycles of 30 min intervals. The solution was desalted (through sequential dialysis using 5 mM, 3 mM, and 1 mM NH_4_HCO_3_) and lyophilized to obtain delipidized, lyophilized FABP3 protein. Before use, lyophilized samples were dissolved in 50 mM Tris-HCl, pH 7.4, and protein samples were quantitated using the Bradford protein assay method (Bio-Rad Protein assay, BIO-RAD).

Human αSyn protein and derivatives (Δ130–140, 130–140CF, and Y133A/Y136A) were purified from BLR(DE3) cells harboring the expression vector pET-SYN, described previously (28). Respective mutations were introduced using the QuikChange site-directed mutagenesis kit. The protocols used to obtain purified αSyn protein were as previously described (60).

### Fluorescence detection of fibril maturation using Thio-T

The formation of αSyn fibrils were detected in fluorescence assays using Thio-T. Samples (1 mg/ml; 69 μM) of either WT or mutant αSyn were incubated in Thio-T assay buffer (50 mM Tris-HCl, pH 7.4, containing 150 mM NaCl and 20 μM Thio-T) with or without additional FABP3 and/or αSyn-derived oligopeptides. One hundred 50 μl aliquots were incubated in 96-well plates (8 × 12-well plate; Greiner) at 37 °C using an ARVOX4 (PerkinElmer) fluorescent plate reader with continuous agitation. The fluorescence signal of the samples (Ex: 440 nm, Em: 486 nm) were measured at 15 min intervals. In experiments where the effects of delayed FABP3 addition were probed (Fig. 1*C*), an equimolar concentration of FABP3 was added to the respective wells after an initial agitation regimen of 3, 8, or 24 h. Data shown are the average of data from three 150 μl samples, and error bars indicate standard errors of the mean.

### TEM measurement

TEM images were taken on a JEOL JEM-1400Plus instrument operating at 80 kV. Ten microliter samples were applied to carbon-coated colloid films coated on copper mesh (400-mesh; Nisshin EM). After application, the sample was allowed to stand for 2 min at room temperature before being blotted off with filter paper. The mesh was washed briefly with 5 μl Milli-Q water, followed by application of 5 μl 10% EM-stainer solution (Nissin-EM). After a 1 min incubation, the stain solution was blotted off, and the grid was again rinsed briefly with 5 μl Milli-Q water. This sample was allowed to dry overnight before analysis.

### AFM measurement

AFM was performed on a Digital Instruments Nanoscope IVa instrument in tapping mode. Various samples that were monitored for changes in Thio-T fluorescence were applied onto freshly cleaved mica and incubated for 1 h at room temperature to deposit fibrillar and aggregated samples. After rinsing the samples with Milli-Q, the sample was allowed to dry at room temperature in air before measurement. The height of the fibrils observed in each sample was measured; the values represent an average of six different measurements, with standard errors.

### CD measurement

CD spectra of samples were measured by taking aliquots of samples used in the respective Thio-T fluorescence assays and diluting these aliquots tenfold with 50 mM Tris-HCl buffer, pH 7.4 to achieve a final protein concentration of 100 μg/ml. These samples were measured on a JASCO J-820 spectropolarimeter in 1 mm path length cells incubated at 25 °C. Spectra represent the average of five scans. Raw spectra were corrected for buffer absorption effects and the presence of FABP3 by subtracting the spectra of these respective components. Averaged CD spectra were analyzed to estimate secondary structural content using BeStSel (http://bestsel.elte.hu/index.php) (27).

### ANS fluorescence assay

The extent of hydrophobicity displayed by FABP3 (2 μM) in the presence of αSyn was estimated by using ANS binding fluorescence assays. Measurements were taken in 50 mM Tris-HCl buffer, pH 7.4. ANS (5 μM) was dissolved in N, N-dimethylformamide before being added to the respective samples. Raw data were corrected for buffer effects by subtraction of a reference spectra.

In certain experiments using the ARVO X4 plate reader, ANS was added to the sample withdrawn from the wells at appropriate intervals. Buffer conditions, as well as the concentration of protein, were identical to the Thio-T assays. However, measurements were taken using an Infinite200 (TECAN) instrument that was capable of measuring fluorescence spectra. The measurement conditions used were an excitation wavelength of 371 nm, and an emission scan from 400 nm to 600 nm.

### Dynamic light scattering

Dynamic light scattering measurements were performed on an Otsuka Electronics FDLS-3000 monitor with a laser wavelength of 532 nm at an observation angle of 90°. Sample protein concentrations were adjusted to 0.3 mg/ml, and a sample volume of 2 ml was used for measurements. Samples were filtered before measurement. Measurements were taken at 25 °C, and 90 scans were averaged to obtain the data and standard error displayed in the figures.

### Quartz crystal microbalance measurement

The binding affinities between αSyn and FABP3 were estimated directly using an Affinix QNμ quartz crystal microbalance apparatus operating at room temperature. Samples of His6-FABP3 were affixed to the gold-plated quartz sensor by nickel chelating binding groups as follows. Before binding, the gold sensor was washed with 1% SDS and piranha solution (H_2_SO_4_:H_2_O_2_ = 3:1). Next, 100 μl of 0.5 mM 3, 3’-dithiobis[N-(5-amino-5-carboxypentyl)propionamide-N’,N’-diacetic acid]dihydrochloride (C₂-NTA, Dojindo) solution was applied and incubated for 10 min at room temperature. This solution was rinsed with Milli-Q water, and 500 μl of Ni+ solution (20 mM Hepes-NaOH buffer, pH 7.5, containing 150 mM NaCl,50 mM EDTA, and 10 mM NiSO_4_) was added. After a 10 min incubation, the sensor was washed again with Milli-Q, then a 0.1 μM sample of His6-FABP3 in 50 mM Tris-HCl buffer, pH 7.4 was applied. The signal from the quartz sensor was then monitored over a 1 s interval with gentle stirring. To initiate the data sampling run, samples of αSyn with varying concentrations were then applied to the sensor. After each experimental run, the bound His6-FABP3 was removed by the application of 500 μl imidazole solution (0.4 M imidazole/20 mM Hepes pH 7.5/150 mM NaCl) for 30 min, followed by a Milli-Q rinse, and reattachment of Ni-ion and fresh His6-FABP3 to reset the sensor to its original state. Analysis of the raw traces to obtain quantitative affinity values were performed using the software package (AQUA 2.0) provided by the manufacturer, according to the following linear relationship between the observed rate constant *k*_obs_ and the concentration of αSyn; *k*_obs_ = *k*_off_ + *k*_on_[αSyn]. Estimated *k*_on_ and *k*_off_ values were then used to determine the K_d_.

### Cell cultivation

Mouse Neuro2a (N2a) cells were obtained from Public Health England. N2a cell cultures were initiated by adding thawed samples in a 37 °C incubator and diluting the thawed cells (1 ml) with 6 ml of minimal essential medium (MEM). This diluted sample was briefly centrifuged for 5 min at 2000 rpm and 4 °C to collect the cell fraction. Ten milliliters of MEM containing 10% fetal bovine serum (FBS) were then added to this fraction, and the cells were incubated at 5% CO_2_, 37 °C to encourage growth. To collect the cells that were grown in this fashion, the cell broth was first aspirated away and then the cells were briefly washed two times with 5 ml phosphate-buffered saline. Next, 5 ml of trypsin-EDTA was added to the wells, and the plates were incubated for 5 min at 37 °C to allow the cells to detach from the wells. Next, 3 ml of 10% FBS+MEM was added to neutralize trypsin activity, and the mixture was centrifuged briefly at 2000 rpm, 4 °C to collect the cell fraction. Pellets were resuspended in 5 ml 10% FBS+MEM, and the cell density was determined using an SLGC cell counting chamber. Based on the cell count, the cell suspension was diluted with 10% FBS+MEM so that the cell density was 1∼5 × 10⁵ cells per ml. This freshly diluted cell suspension was then applied in 400 μl aliquots for a new round of cell culture.

### MTS assay

The ratio of live-to-dead cells in a given cell culture was estimated using the MTS assay. Forty eight-well plates containing cell cultures that had been cultivated to 80∼90% confluence were washed by the addition of 200 μl phosphate-buffered saline/well. Next, MEM media was added to each well (400 μl/well), and the plates were incubated for 24 h at the indicated experimental conditions. After this initial cultivation time, samples containing various combinations of αSyn and/or FABP3 was added to the culture, and incubation was allowed to proceed for an additional 24 h. Finally, 80 μl MTS solution (Promega) was added to each well, and the absorbance of each sample was monitored at 490 nm using a SpectraMax M2^e^ plate reader to estimate cell viabilities.

### FRET experiments using cultured cells

FRET signals between GFP-αSyn and FABP3-mCherrry expressed in N2a cells were observed using a confocal laser microscope (FLUOVIEW FV10i, Olympus, GFP Ex: 489 nm/Em: 510 nm, mCherry Ex: 580 nm/Em: 610 nm). FRET measurement conditions were set to be identical in emission sensitivity and contrast to enable comparison. Cells expressing both GFP-αSyn (or GFP-αSynΔ130-140) and FABP3-mCherrry were grown under 5% CO_2_ at 37 °C for 48 h after introducing plasmids pCAG-GFP-αSyn (or pCAG-GFP-αSyn Δ130-140) and pCAG-FABP3-mCherry. Introduction of plasmid into N2a cells cultivated to 80∼90% confluence in 10% FBS+MEM was performed according to the manufacturer’s protocols using Lipofectamine 3000 Transfection Reagent (Thermo Fisher Scientific). For the FRET experiments in the presence of αSynP130-140, 50 μM peptide was added at the same time of the addition of plasmids. Nuclear staining was performed with Cellstain Hoechst 33342 solution (Dojindo), and cells were immobilized with 4% paraformaldehyde. SlowFade Diamond Antifade Mountant (Thermo Fisher Scientific) was dropped on the slide glass to suppress fading of the fluorescence signal. Statistical comparisons were performed by Welch’s *t* test.

### Cytotoxicity assay expressing αSyn and FABP3 in cells

Cell viability measurements monitored by mitochondrial stability were assayed by measurement of mitochondrial membrane potential. In this experiment, pCAG-αSyn (or pCAG-αSyn Δ130-140) and pCAG-FABP3 were introduced as described in the FRET experiment section above. For experiments involving the presence of αSynP130-140 in culture, 50 μM peptide was added at the same time as the time of addition of plasmids. The resultant cells were incubated for 6 h in the presence of 1 μM rotenone and stained with 10 μM 5, 5’, 6, 6’-tetrachloro-1, 1’, 3, 3’-tetraethylbenzimidazolylcarbocyanine iodide (JC-1; AdipoGen Life Sciences) for 10 min at 37 °C. A confocal laser microscope (FLUOVIEW FV10i, Olympus, JC-1 monomer Ex: 489 nm/Em: 510 nm, JC-1 aggregate Ex: 578 nm/Em:598 nm) was used to visualize mitochondrial viability. Conditions for measuring JC-1 monomer and JC-1 aggregate fluorescence were set to be identical in excitation power strength, emission sensitivity, and contrast to enable comparison.

Cell viability measurements using a Tali Image-Based Cytometer (Thermo Fisher Scientific) were performed according to previous methods (59). After 24 h incubation, media was changed to 0% FBS+MEM containing 30 μM 6-hydroxydopamine and incubated for 24 h. Statistical comparisons were performed by Welch’s *t* test.

## Data availability

All data are presented in the article. All of the data that were used to produce the figures in the present study are available upon request from Yasushi Kawata, Tottori University (kawata@tottori-u.ac.jp).

## Conflict of interest

The authors declare that they have no conflicts of interest with the contents of this article.
